# Silicon-Based Technologies for Flexible Photovoltaic (PV) Devices: From Basic Mechanism to Manufacturing Technologies

**DOI:** 10.3390/nano11112944

**Published:** 2021-11-03

**Authors:** Sangmo Kim, Van Quy Hoang, Chung Wung Bark

**Affiliations:** 1 School of Intelligent Mechatronics Engineering, Sejong University, Seoul 05006, Korea; singmul0227@gmail.com; 2 Department of Electrical Engineering, Gachon University, Seongnam 13120, Korea; quybk20113989@gmail.com

**Keywords:** photovoltaic, silicon, flexible, energy conversion

## Abstract

Over the past few decades, silicon-based solar cells have been used in the photovoltaic (PV) industry because of the abundance of silicon material and the mature fabrication process. However, as more electrical devices with wearable and portable functions are required, silicon-based PV solar cells have been developed to create solar cells that are flexible, lightweight, and thin. Unlike flexible PV systems (inorganic and organic), the drawbacks of silicon-based solar cells are that they are difficult to fabricate as flexible solar cells. However, new technologies have emerged for flexible solar cells with silicon. In this paper, we describe the basic energy-conversion mechanism from light and introduce various silicon-based manufacturing technologies for flexible solar cells. In addition, for high energy-conversion efficiency, we deal with various technologies (process, structure, and materials).

## 1. Introduction

Over the past decade, the global energy crisis, while subject to traditional problems such as global warming, ozone depletion, carbon emissions from fossil fuels, and decreasing availability of fossil energy (oil, gas, and coal), is facing new challenges [1,2,3]. For example, newly developing countries require much more energy for their growth. Future technology, such as the Internet of Things (IoT), will also require more energy. Therefore, renewable-energy technology is essential to enable further energy production [4]. To date, many non-renewable energies are produced in limited amounts in specific parts of the world and take a long time to replenish, making energy more abundant for some nations than others. In contrast, renewable energy sources are found anywhere on Earth, making them more abundant depending on know-how and technology [5,6]. All nations are given an amount of energy from sun, wind, plant, and geothermal sources for free. Among the various types of renewable energy sources, photovoltaic (PV) technology using Sol. Energy has been more developed than other sources. From the sun, approximately 1.7 × 10^17^ W of Sol. Energy irradiates the Earth, which is more than 10,000 times the global energy consumption [6,7].

Conventional PV cells are made from a silicon wafer that transforms sunlight directly into electricity. These silicon-based solar cells use 150 to 200 μm crystalline silicon wafers, which are often brittle and hard [8]. Therefore, niche flexible PV-cell applications have been developed using diverse methods, such as low-temperature and solution processes with thin-film materials deposited on flexible substrates. Despite being flexible, light, and thin, they have a short lifetime, low energy-conversion efficiency, and a small active area, and include harmful materials. Silicon-based PV cells can become bendable or flexible when silicon wafers are sufficiently thin. Flexible PV cells with a silicon substrate can work much better than other similar flexible materials [9,10].

In this study we consider a basic mechanism for the conversion from Sol. Energy to power generation and the progress in PV development by using silicon materials. We consider only flexible, lightweight, and thin PV devices using silicon-based elements. Finally, we provided a summary of the prospective development and technology related to flexible silicon-based solar cells.

## 2. Basic Theory for the Conversion from Sol. Energy to Power Generation

### 2.1. Basic Photoconversion Mechanism and Structure

**Figure 1:**
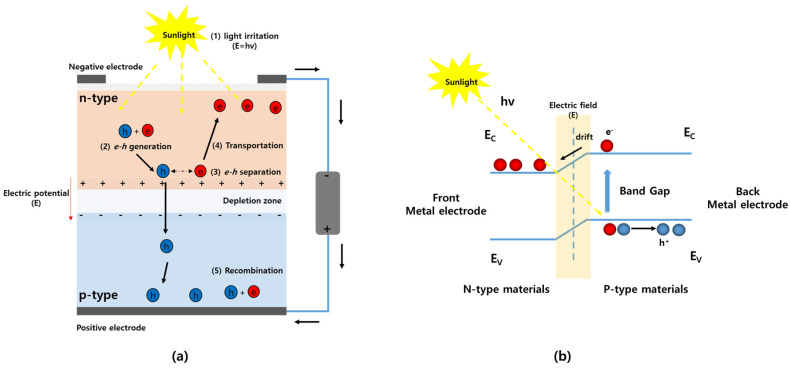
(**a**) working principle of solar cell with p-n junction structure and (**b**) loss mechanism in standard p-n junction solar cells.

In this section, we introduce a simple structure of a solar cell and discuss its operating process under sunlight. Electron–hole pairs should be successfully separated and prevented from recombining in order to achieve solar-energy conversion. Therefore, selecting materials that are efficient in absorbing Sol. Energy (photons) can accelerate electrons to high energies and transfer them to an external load through an electrical circuit is crucial. A basic solar cell is designed with p-n junction semiconductor materials that convert the energy of light into direct current (DC) electricity using the PV effect. The p-n junction semiconductor consists of two types of semiconductor materials: a negative (n)-type material and a positive (p)-type material, as shown in Figure 1a [11]. P- and N-type materials are used for commercial silicon material; their polarity is created by doping the material with phosphorus (P) or boron (B). As electrons and holes are diffused to the opposite low-charge concentration of each material, the depleted region is naturally created around the junction interface. The depletion layer is typically small, and the electric field E is present inside the depleted region without electron–hole recombination. The electron–hole pairs are generated inside the junction’s depleted region by photon energy from sunlight. Assuming uniform illumination inside the depleted region, electricity generation from sunlight involves four steps [11,12]:

1. Light absorption and generation of carriers. Photons originating from sunlight arrive at the surface of the solar cell, which absorbs them. Many electron–hole pairs are produced by the photon-absorption mechanism.
2. Carrier separation and collection of the light-generated carriers to generate a current. The electrons are released into the negative layer, and the decomposed electrons flow through the positive layer.
3. Generation of a voltage across the solar cell. The electrons overcome the boundary energy at the n-type layer and flow through the negative electrode at the top of the cell, which is connected to an external load. This provides a path for the positive material layer; thus, electricity is generated. When the electric field is sufficiently strong to cease further diffusion of holes and electrons, the depleted region reaches equilibrium. Integrating the electric field across the depleted region determines what is called the built-in voltage (also called electric field or voltage). Electrons and holes diffuse into regions with lower concentrations of them.
4. Carrier collection and dissipation of power in the load. Electrons return to the cell after exiting the external load and repeatedly move from the positive to the negative side for continuous electricity generation.

Because of the built-in potential of p-n junctions, the minority carriers (electrons in p-region move towards the n-region, holes in the n-region move toward the p-region) are separated as shown in Figure 1a. These minority charge carriers are collected by metallic contacts. The common unit cell of a single-junction silicon solar cell can produce an open-circuit voltage (*V_OC_*) of approximately 0.6 volts. To increase voltage to be stably applied for various electrical applications, solar cells are combined into a large panel with serial and parallel connections of the unit cells, so that a great deal of electricity can be generated [12].

Figure 1b shows the loss mechanisms in standard PN-junction solar cells. The usual mechanism is that absorption of a photon generates an electron–hole pair. In other words, under illumination, photogeneration results from an interaction of an electron with a photon; the electron is promoted into the conduction band, leaving behind a hole. However, if the electron doesn’t absorb enough energy from light, it cannot be exited from the valence band (VB) to the conduction band (CB). Solar cells eventually cannot convert photons with energy less than the energy band gap into electricity. Moreover, at least 50% of the incident Sol. Energy would be lost. Other loss factors include non-absorption/reflection, junction loss, contact loss, and recombination loss. Even though the same amount of light shines on a solar cell, the energy-conversion efficiency of solar cells is considerably below what was expected [13].

### 2.2. Performance Evaluation of Solar Cells

The exciton has enough energy to move an electron to generate electricity when the following processes occur: (1) light absorption and exciton formation; (2) exciton dissociation into free charge; (3) free-charge transport and collection; (4) free-charge recombination into excitons, and (5) exciton annihilation. As mentioned above, the total power-conversion efficiency (PCE) of a solar cell depends on process steps 1–4. Quantum efficiency (QE) should be considered to understand the PV mechanisms in solar cells. QE is one of the most important parameters used to evaluate the performance of solar cells. It depends on the spectral response to reflect its wavelength dependence [11,14]. QE normally includes all energy loss of the solar cell itself by the absorbance reflection and transmittance in incident light. The QE of a solar cell could be evaluated by External Quantum Efficiency (EQE) or Internal Quantum Efficiency (IQE). EQE is the ratio between the charge carriers collected by the solar cell and the photons given energy on the solar cell by incident photons. In other words, it means how the solar cell converts electrons to photons. The EQE focuses on the number of carriers collected on a solar cell from solar-energy photons. EQE is called the Incident photon-to-electron conversion efficiency (IPCE). The total value of EQE (η_ext_) can be calculated using the following equation [14]:

η = η_absorption_ × η_exciton_ × η_collection_

where η_absorption_ is the light-absorption efficiency, η_exciton_ is the exciton-dissociation efficiency (including exciton transport), and η_collection_ is the charge-collection efficiency. If spectral responsivity (SR) is introduced (SR: Ratio of the photocurrent generated by the solar cell and the power incident):

EQE = (hc/qλ) × (SR)

where h is Planck’s constant, c is the speed of light, q is the electronic charge, and λ is the wavelength. IQE is called the quantum yield and can be calculated by the recombination rate, that is, how many electrons and holes are recombined in the active layer of a solar cell. IQE is higher than EQE because all irradiated light is not absorbed in the active layer and all photogenerated electron–hole pairs are generated out of the solar cell [15]. After EQE of a solar cell is measured, IQE can be calculated with EQE data combined with transmission and reflection using the following equation:

IQE = (number of electrons)/(number of absorbed photons) = EQE/(1 − R)

where T is transmission and R is reflection. The parameters for performance of a solar cell are evaluated by short-circuit current, open-circuit voltage, fill factor, and conversion efficiency [15].

(a) Short-Circuit Current (*I_SC_*)

Short-circuit current (or short-circuit photocurrent) is the current that flows through a solar cell when the voltage applied across the solar cell is close to zero. In other words, when a solar cell is short circuited, the current is that flowing through the solar cell, and its value is negative. In a solar cell, maximum short-circuit current depends on various factors; these are device area, light intensity, spectral distribution of incident light, and absorption/reflection on the surface of the solar cell. To get a high short-circuit current (ISC), the number of electrons and holes that shift to each electrode should increase without photogenerated electron and hole recombination and be prohibited from reflecting on the surface of the solar cell [15,16].

Solar cells with p-n junctions, such as a diode, have non-linear current–voltage (I-V) characteristics. The I-V curve of a solar cell is the superposition of the I-V curve of the solar-cell diode in the dark with the light-generated current IL. The light shifts the I-V curve down into the fourth quadrant, where power can be extracted from the diode. The equation for the I-V curve in the first quadrant is [17]:

$$I_{dark}=I_{0}\left[e^{\frac{qV}{KT}}-1\right]$$

$$I=I_{SC}-I_{dark}=I_{SC}-I_{0}\left[e^{\frac{qV}{KT}}-1\right]$$

where is *I* is total current, *I_D_* is diffusion current, *I_L_* is light-generated current, *I*_0_ is a constant, *q* is electronic charge, *K* is Boltzmann’s constant, and *T* is absolute temperature (°K).

$$J_{SC}=qG\left(L_{n}+L_{p}\right)$$

where *q* is the elementary charge, *G* is the generation rate, and *L_n_* and *L_p_* are the electron and hole diffusion lengths, respectively. If the diffusion length of the charge carrier is smaller than the thickness of the cell, the short-circuit current would decrease.

To remove the influence of the solar-cell area, the short-circuit current density (*J_SC_*, mA/cm^2^) is used; it is measured from the standard 100 mW/cm^2^ of the solar spectrum (AM1.5). Short-circuit current (*I_SC_*) is the maximum current and is calculated as follows [17]:

$$I_{SC}=\int_{0}^{\infty }J_{SC}\left(\lambda \right)d\lambda ≅\int_{0.3\mu m}^{\lambda_{0}}J_{SC}\left(\lambda \right)d\lambda$$

where λ is wavelength from incident light. Typical silicon-based solar cells under an AM1.5 spectrum have a maximum current density of 46 mA/cm^2^.

(b) Fill Factor (*FF*)

*FF* represents how easily the device produces the carriers that are photogenerated by light absorbed inside the solar cell. The theoretical value of *FF* is 100%, close to the rectangular shape shown in the graph of the I-V curve. Unfortunately, *FF* cannot reach 100%. Even in the reported solar cells, it is limited to a maximum *FF* of 90%. Typical *FF* values are distributed in the range of 0.7 to 0.8. There are several factors that can significantly influence *FF*, and these factors interact with each other very intricately. As light power incident on the device, *FF* is expressed by [16,17]:

$$\mathrm{Fill}\;\mathrm{Factor}=\frac{P_{m}}{I_{SC}}=\frac{I_{m}V_{m}}{I_{SC}V_{OC}}$$

where *I_m_* is the maximum power current and *V_m_* is the maximum power voltage on the external load.

**Figure 2:**
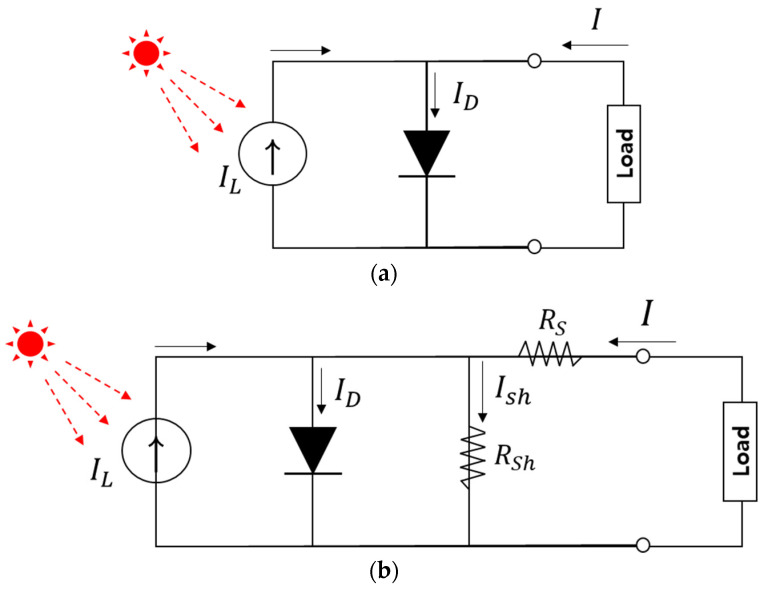
Equivalent electrical circuit of (**a**) an ideal solar cell and (**b**) an actual solar cell.

Figure 2 shows the equivalent electrical circuit of a solar cell. Conventional solar cells consist of a current source (the light-generated current) and a diode with a dark saturation current. No series resistance and no shunt resistance are present. If *R*_S_ (series resistance) and *R*_SH_ (shunt resistance resistance) have no effect on the performance of the device, the fill factor can be redefined as:

$$\mathrm{Ideal}\;\mathrm{Fill}\;\mathrm{Factor}\;\left(FF_{0}\right)=\frac{V_{OC}-\ln\left(v_{OC}+0.72\right)}{v_{OC}+1}$$

**Figure 3:**
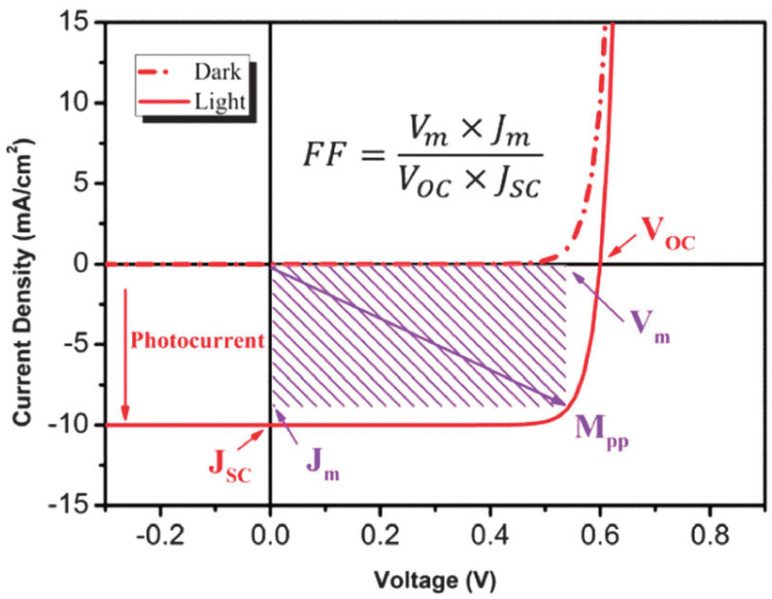
Typical current-voltage (I-V) characteristics for dark and light current in a solar cell, illustrating the important parameters for such devices. *J_SC_* is short-circuit current density, *V_OC_* is open-circuit voltage, *J_m_* and *V_m_* are the current density and voltage at the maximum power point (Mpp), and *FF* is the fill factor. Reprinted with permission from ref. [17]. Copyright 2013 Royal Society of Chemistry.

Moreover, *FF* can be defined amount by which *J_m_* and *V_m_* rectangle is filled as the relationship between dark and light current in typical current–voltage (I-V) characteristics for dark and light current in a solar cell, illustrating the important parameters for devices as shown in Figure 3.

**Figure 4:**
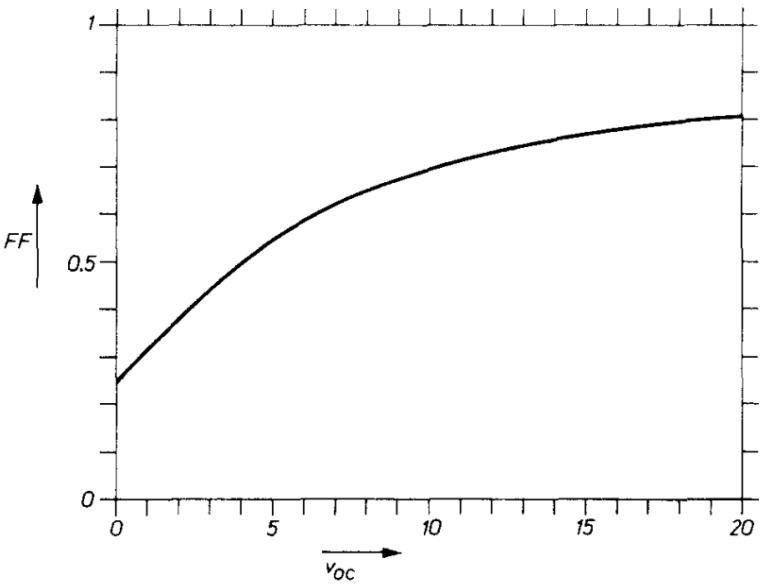
Dimensionless open-circuit voltage and its relationship with *FF*. Reprinted with permission from ref. [16]. Copyright 1983 Elsevier.

We introduce the result only in the form of an *FF*-*v_OC_* curve in Figure 4, where *v_OC_* is the dimensionless voltage *v_OC_ = qV_OC_/kBT*. *FF* is increasing as *v_OC_*.

In practical solar cells, parasite resistances are unavoidable factors. Therefore, the series resistance and shunt resistance are introduced into the equivalent model to account for these energy losses (Figure 3). Usually, series resistance is derived from the bulk resistances of the active layer and electrodes, and the contact resistances between the active layer and electrodes. The shunt resistance originates from the current leakage induced by the pinhole in the cell, or the current leakage from the edge of the device.

(c) Power-Conversion Efficiency (PCE)

PCE is an essential characteristic that determines the energy-conversion efficiency of a solar cell. The efficiency is defined as the ratio of energy output from the solar cell to input energy from the Sun. The efficiency (η) measured at standard dark and illumination conditions (AM 1.5: 100 mW/cm^2^ at 25 °C) show the parameter of electricity generation to compare the performance of solar cells:

$$\eta =\frac{P_{out}}{P_{in}}=\frac{I_{SC}\times V_{OC}\times FF}{P_{in}\times A}$$

## 3. Technology for Improving the Power-Conversion Efficiencies

Traditionally, silicon-based solar cells are limited to approximately a 29% power-conversion efficiency. Sunlight has many kinds of wavelength (ultraviolet, infrared, visible, etc.). Even though all light could be to be changed to more useful energy such as electricity, a silicon-based solar cell absorbs only light of 400~1000 nm because of the 1.11 eV energy band of silicon. Other light, that is, short wavelengths (under 500 nm) or long wavelengths (above 1000 nm), cannot be absorbed and is thrown away. Therefore, we should focus on how much light is used.

**Figure 5:**
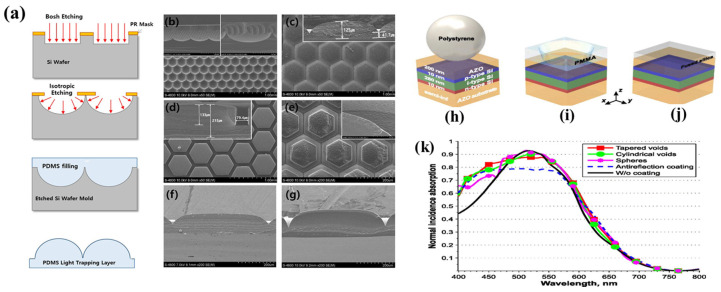
(**a**) Schematic of the fabrication of light-trapping layers, from etching the silicon (Si) wafer to molding the polydimethylsiloxane (PDMS). Scanning electron microscopy (SEM) images of the (**b**) Si wafer master mold in plane and cross-sectional view and light-trapping layers with a (**c**) lens array; (**d**) pillar-shaped lens array; (**e**) lens array with rough surfaces (inset: cross-sections); (**f**) flat-topped lens array, and (**g**) lens array with spaces between patterns. Reprinted with permission from ref. [18]. Copyright 2015 AIP Publishing. (**h**–**k**) Schematic views of thin-film solar cell with three types of all-dielectric coatings for TFSC: (**h**) a close-packed layer of dielectric (polystyrene) nano- or microspheres; (**i**) a square array of tapered nanovoids in the superstrate, (**j**) a homogeneous film ARC, and (**k**) absorption spectra of the system for the following cases. Reprinted from ref. [19].

First, efficient capture of light is essential for improving the efficiency of solar cells. As mentioned above, because only part of the light can be used, it is important for the solar cell to be designed to minimize optical losses itself. For improving light absorption in solar cells, technologies such as anti-reflective coating and dielectric and light-trapping coating have been introduced, as shown in Figure 5. In Figure 5a–g, the light-trapping layers were built on the device via dry etching with a pattern of the PR (photoresist) as the designed mask on the silicon substrate and molding with polydimethysiloxane (PDMS) for a lens array, pillar array, and lens array with rough surfaces. Actually, this technique was applied for improving the efficiencies of Dye Sensitized Solar Cells (DSSC), and their energy-conversion efficiencies increased to 70% over that of the cell without light-trapping layers. Figure 5h–k shows the result of non-resonant all-dielectric coatings for increasing the photovoltaic absorption. P. M. Voroshilov et al. prepared three different all-dielectric structures (a flat anti-reflecting coating, an array of densely packed polystyrene nanospheres, and an array of nanovoids, both cylindrical and tapered shapes, in the PMMA layer) on the amorphous silicon-based solar cell. Among them, the highest light-trapping property was shown by the nanovoids (Light-trapping efficiency, LTE: Tapered voids 2.57, cylindrical voids 5.04, spheres 2.54, ARC 1.31).

Second, in terms of effective carrier generation, photogenerated carriers (electron–hole) are easily separated in the internal electric field, and carriers should be diffused at short times in solar cells. For the effective electron–hole pairs to generate and separate as induced by photogeneration, the valence band (VB) and the conduction band (CB) of the semiconductor materials that consist of p type and n type are important factors. These are calculated using the following equations:

$$E_{\mathrm{VB}}=\chi -E_{C}+0.5\left(E_{g}\right)$$

$$E_{\mathrm{CB}}=E_{\mathrm{VB}}-E_{g}$$

where *E*_VB_ and *E*_CB_ are the valence (VB) and conduction (CB) band edge potentials, respectively; χ is the electronegativity value, which is the geometric mean of the electronegativities of the constituent atoms (the electronegativity of an atom is the arithmetic mean of the atomic electron affinity and the first ionization energy); *E*_C_ is the energy of free electrons on the hydrogen scale (~4.5 eV), and *E_g_* is the bandgap energy of the semiconductor. For tuning the bandgap, impurity atoms used as dopants, such as boron (B), phosphorus (P), and arsenic (As), occupy substitutional positions where the dopant atoms can contribute free electrons or holes to the silicon lattice of pure silicon.

If the carriers recombine, then the electron-hole (e–h) pair is lost, no photocurrent can flow in the device, and power is not generated. The recombination of photogenerated e–h pairs could occur at the surfaces of cells and at the back counter electrode of solar cells because of the inherent defect and trap level in the bandgap. To reduce losses by recombination, a passivating contact layer is required between the silicon wafer and the metal terminals contacted with the external load.

**Figure 6:**
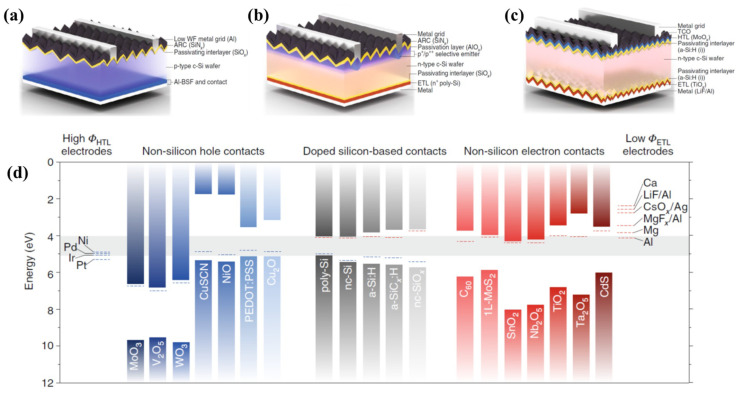
Solar cells featuring passivating contacts. (**a**) Hybrid metal–insulator–semiconductor (MIS) cell; (**b**) electron tunnel-oxide passivating contact (TOPCon) solar cell; (**c**) dopant-free asymmetric heterocontact (DASH) solar cell; (**d**) materials for passivating contacts. Reprinted with permission from ref. [20]. Copyright 2019 Springer Nature.

Commercial silicon-based solar-cell manufacturing goes through many processes, such as front-surface texturing, phosphorus diffusion (p-n junction), passivation film deposition, anti-reflective layer coating, electrode patterning (screen printing), and metal contact. Figure 6a–c shows various kinds of solar cells with an inserted passivation layer. The front-side texture is an essential layer, because a solar cell with an untreated surface reflects over 40% of the light because of the smooth surface. The addition of the passivation layer in the solar cell helps prevent recombination, could reduce the defect, and thus increases the device voltage. The hybrid metal–insulator–semiconductor (MIS) cell (see Figure 6a) is a representative crystalline silicon solar cell (c-Si solar cell) with passivating contacts. The tunnel-oxide passivating contact (TOPCon) solar cell (Figure 6b) has a structure that features a front-side boron (B+)-diffused selective emitter for the hole contact and a rear phosphorus-doped poly crystalline silicon (p-Si) electron contact. A dopant-free asymmetric heterocontact (DASH) solar cell is applied for the dopant-free metal oxide and fluoride electron and hole transport layers to replace the doped silicon layers.

For the insertion of passivating thin films, candidate materials for passivating contacts were introduced (Figure 6d). Metal oxides (aluminum oxide (Al_2_O_3_), silicon oxide (SiO_x_), silicon nitrite (SiN_x_), and hydrogenated amorphous silicon (a-Si:H), etc.) have been deposited via a vacuum deposition method, such as by magnetron sputtering. Recently, solar cells with a dopant-free concept have been emerging because of their simpler preparation and low temperature.

The traditional solar cell with a p-n junction has been replaced by a solar cell with a dopant-free heterojunction, which depends on the work functions of materials that do not require additional doping. The most-studied dopant-free materials are based on metal oxides, as shown in Figure 6. The inserted passivation layer has also been playing an increasingly important role in anti-recombination activity. Therefore, it is very important to select materials for optimal design of devices to improve the photovoltaic performance.

Next, we explain the role of the resistance in a solar cell. The resistance of a device consists of two kinds: series resistance (*R*_S_) and parallel (shunt) resistance (*R*_SH_), as shown in Figure 2. The resistance of materials has a direct effect on the efficiencies of the device and a decrease in their value leads to a loss of the solar cell itself. For an ideal solar cell, *R*_S_ is 0 ohm and *R*_SH_ is close to infinity. As the shunt resistance of a solar cell increases, the active area (power generation) shrinks, and the point of maximum *V_OC_* and *I_SC_* is approaching zero from the ideal slope of the I-V curve, as shown in Figure 3, including the degradation of the fill factor. Series resistance is the resistance of materials between two electrical contacts (terminals), as in Figure 2a, in addition to the contact resistances of the front and back electrodes that connect the load. All electrode materials have their own resistance. The opaque metal materials used are Al, Au, Pt, Cu, and Ag (<10^−5^ ohm-cm), and transparent electrode materials (transparent conducting oxides) include tin-doped indium oxide (ITO, <10^−4^ ohm-cm) and fluorine-doped tin oxide (FTO, <10^−4^ ohm-cm). ITO have been used as material for transparent electrodes with more than 90% optical transmittance in the visible range. However, compared to FTO, ITO is more sensitive to heating; its own resistance increases as temperature on the device increases. High temperatures cause higher resistance and contribute to efficiency losses in the solar cell. Moreover, a transparent electrode is related to the active layer of the solar cell. The larger the area, the better, but it shows the problem of increasing the own resistance at that time. The selection of its quality is important for improving the power-conversion efficiencies.

## 4. Technology of Ultrathin Silicon for Flexible Solar Cells

Silicon wafers are divided into crystalline (mono- and poly-) and amorphous silicon. Conventional manufacturing processes for solar cells have employed thick Si wafers of 100–500 μm. Because of the hardness and brittleness of normal silicon wafers, such silicon-based solar cells are incompatible with flexible devices for bending and being lightweight. Recently, an ultrathin silicon wafer has been developed. Its thickness was dramatically shrunk for flexibility suitable for covering and bending the surface [21,22].

**Figure 7:**
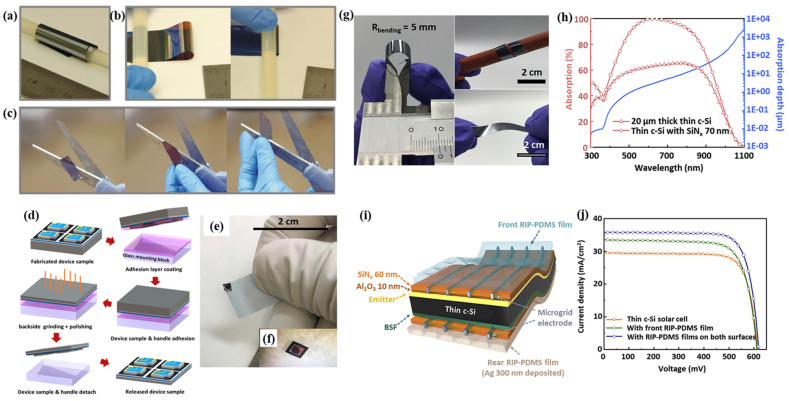
(**a**,**b**) Ultrathin silicon films (~3 μm) that are flexible and bendable; (**c**) photo images when cutting the silicon films. Reprinted with permission from ref. [24]. Copyright 2013 American Chemical Society; (**d**) sample fabrication process to release the ultrathin crystalline silicon (c-Si) cell from the substrate; (**e**) photos of the free-standing c-Si cell (total sample thickness, 8 μm; cell thickness, 2.7 μm); (**f**) photo of the free-standing c-Si cell attached to a quartz wafer for testing in a solar simulator. Reprinted with permission from ref. [23]. Copyright 2020 Elsevier; (**g**) photos of an ultrathin c-Si substrate (thickness ~20 μm); (**h**) absorption spectra (left *y* axis) of ultrathin c-Si with and without a silicon nitrite (SiN_x_; thickness, ~70 nm) layer and the absorption depth (right *y* axis) of the c-Si substrate; (**i**) schematic of a thin c-Si solar cell and (**j**) current density–voltage (J-V) characteristics of thin c-Si solar cells with and without RIP-PDMS films. Reprinted with permission from ref. [22]. Copyright 2020 Elsevier.

The greatest advantage of an ultrathin substrate is that it can be applied to a fully bendable surface. As shown in Figure 7a,b, the Si film is 3 μm thick and can be wrapped around a plastic rod (diameter 7 mm). In addition, the films can be unfolded and folded to a radius of around 1 mm. Films are easily cut with normal scissors along the crystalline directions (Figure 7c). For thinning a film, mechanical polishing is used, as shown in Figure 7d. M. Xue et al. [23] successfully reduced a bulk Si wafer (~500 μm) to a thickness of 2.7 μm via grinding with aluminum oxide grit (particle size, 9 μm) and polishing with colloidal silica. A flexible ultrathin crystalline silicon solar cell has a total sample thickness of 8 μm and efficiency above 12.0%, as shown in Figure 7e,f [24].

## 5. Cell Manufacturing from Materials

### 5.1. Device Manufacturing Methods

(a) Roll-to-Roll Printing

**Figure 8:**
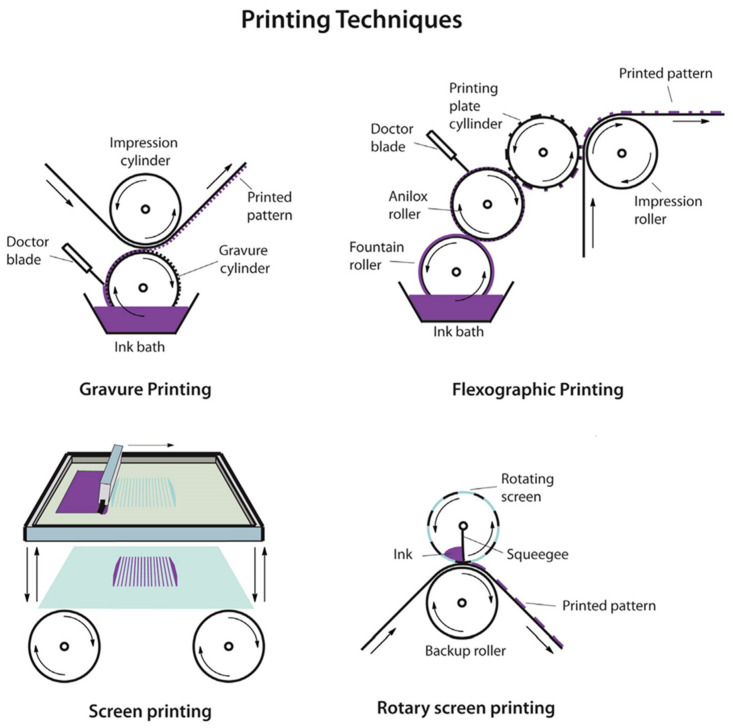
Illustrations of four printing techniques: gravure printing, flexographic printing, screen printing, and rotary screen printing. Reprinted with permission from ref. [27]. Copyright 2012 Elsevier.

Flexible PV devices cannot be manufactured using general PV device processes that involve high-vacuum film deposition and high temperatures. Therefore, new manufacturing methods are required. In many reports about flexible PV devices, roll-to-roll (R2R) processing has been highlighted as the latest high-throughput, large-scale production technology [25]. R2R processing is associated with printing techniques. Figure 8 shows four representative printing methods.

First, gravure printing is widely used for printing magazines and high-volume print runs, such as catalogs. Successful gravure printing depends on the uniformity of the ink transferred to the patterned substrate. A gravure printing machine consists of two heavy cylinders, a doctor blade, and an ink supply. The substrate is fed between the top and bottom cylinders and is printed with the desired pattern. Then, the doctor blade removes excess ink. The technique was developed and used for photographs and newspapers in the past, and its potential has been reported in a few journals about solar-cell production [26,27].

However, flexographic printing is considered to be faster and simpler than gravure printing [26]. Flexographic printing involves directly printing rubber or plastic materials, such as plastic polyvinylidene fluoride or polyvinylidene difluoride (PVDF). Flexographic printing has been used for printing transparent electrodes and active layers of organic solar cells. However, the quality of the finished product is poor, despite its advantages, owing to the low pressure used to apply the ink.

Screen printing is the most popular printing technique and has been used for more than 100 years in the design of fabrics, paper, and ceramics. The technique involves a mesh stretched over a modified frame and uses a squeegee to manually transfer ink onto the overlapped substrate. Screen printing enables the quick formation of a layer on the substrate, which is a very low-cost process.

Rotary screen printing employs an automatic roller, rather than the manual squeegee used in conventional screen printing, which can quickly print a larger area with a more uniform layer than that from manual screen printing [27].

(b) Sputtering

Among the film-deposition techniques, sputtering is the best option for the preparation of large-scale high-quality films. Sputtering requires a vacuum chamber to maintain a very low pressure (<30 mTorr) working gas (argon, oxygen, nitrogen, etc.) that affects the target. The gas inside the discharged plasma bombards the target and sputters off the materials that are deposited on the substrate. However, when sputtering is applied, high-energy particles, such as Ar, negative ions, and secondary ions with high kinetic energy, directly bombard the substrate and film material. Consequently, films prepared by sputtering have defects in the crystal structure, have low density, and suffer from the disorder in an ion bombardment crystal caused by internal stress. Moreover, the temperature of the substrate increases to over 100 °C, which is unsuitable for polymer substrates [28].

**Figure 9:**
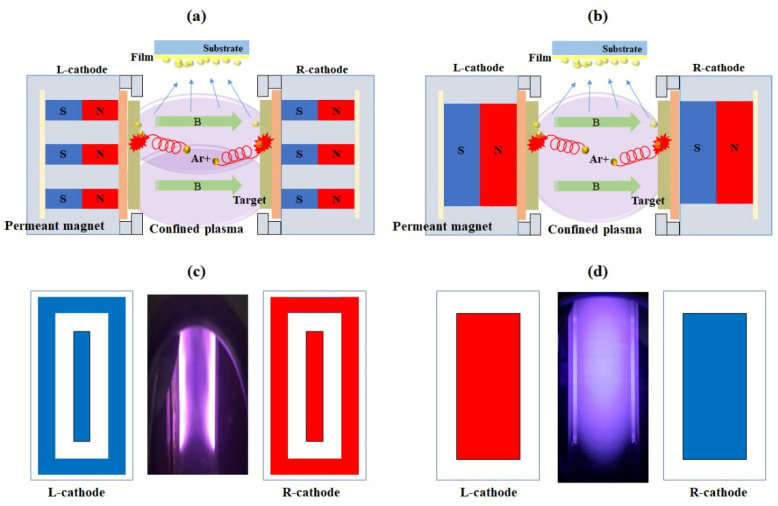
Schematic of the facing target sputtering system with various magnet arrays: (**a**,**c**) Open-type FTS, (**b**,**d**) Closed-type FTS, and plasma confined between targets for low-temperature sputtering. Reproduced with permission from ref. [29].

Recently, facing target sputtering (FTS) has been introduced to address problems encountered by conventional sputtering. During film deposition in the sputtering system, high-density plasmas are induced by the working gas, and electrons, as well as secondary and charged ions, undergo a round-trip helical motion between the two cathodes. The substrate is placed above the two targets that are outside the plasma stream, as shown in Figure 9. Thus, FTS allows film deposition in a low-temperature atmosphere. Kim et al. [29] investigated the substrate temperature during film sputtering. The temperature was increased to 80 °C after sputtering for 1 h in the FTS system. FTS can control plasma confinement by controlling the cathode installed inside the magnetic-field arrangement of the system to ensure that the quality of the film can be improved under a lower-temperature environment. Jeong et al. [30] reported that Al-doped ZnO (AZO) films with a sheet resistance of 39 ohm/sq (film thickness ~330 nm) and average transmittance of 84.86% were deposited on a polyethylene terephthalate (PET) substrate using FTS.

(c) Spin Coating

**Figure 10:**
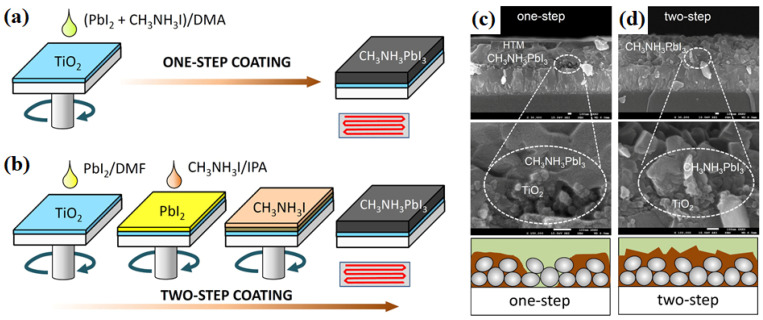
One- and two-step spin-coating procedures for CH_3_NH_3_PbI_3_ formation and cross-sectional SEM images of (**a**,**c**) one-step, and (**b**,**d**) two-step deposition of CH_3_NH_3_PbI_3_. Reprinted from ref. [32].

Spin-coating techniques are the most appropriate film preparation methods for flexible PV devices. Normally, spin coating is performed at room temperature. Materials (typically liquids) are dropped onto the center of a rotating substrate driven by a mechanical vacuum motor. The film thickness is defined by the rotational speed, acceleration, spinning time, and drying conditions. The most important condition for defining the film thickness is the number of coats applied, whereas the film properties depend on the drying temperature after the spin process [31]. Im et al. [32] reported the effects of spin coating on PSCs. After the TiO_2_ layer was applied to a fluorine-doped tin oxide (FTO) substrate, PbI_2_ and CH_3_NH_3_I were deposited on the compact and mesoporous TiO_2_/FTO substrate using either a one- or a two-step method (Figure 10). Unlike that produced using the one-step method, a CH_3_NH_3_PbI_3_ film prepared using the two-step method was formed without defects or voids. In addition, the PCE increased from 7.5 to 13.9% for the two-step-deposited cells compared to that of the one-step cells.

(d) Spray Coating

Spray-coating technology has recently attracted attention as a large film-deposition method, rather than spin coating, which is the best option for film preparation in high-performance organic PV solar cells, such as perovskite solar cells (PSCs). However, in terms of scalable deposition, spin coating cannot cover large thin films on the substrate to the industrial scaling level. Spray coating has been used to grow thin films at low temperatures. It has many advantages, such as a relatively simple, low-cost deposition technique, R2R, and film covering on a large-area substrate. Moreover, it is a high-speed film deposition process because it can dry quickly [33,34].

**Figure 11:**
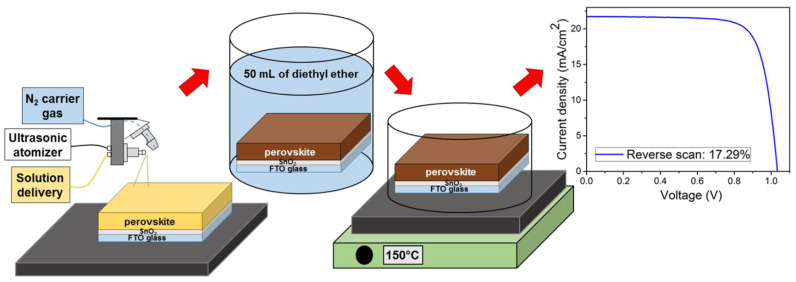
Fabrication of perovskite solar cells (PSCs) with spray-coating and PSC samples. Reprinted from ref. [35].

As shown in Figure 11, a conventional spray-coating system consists of a hopper that supplies solutions, a nozzle (driven by high-pressure air or nitrogen or ultrasonically), and a solution supply tank. After the solution in the supply tank passes through the stream nozzle, it breaks down into ultrafine droplets, and a continuous flow can be dispensed directly onto the substrate. After covering the substrate with the dispensed droplets, the solvents were dried by heating to form a film. The film thickness can be controlled based on the solution conditions, status of the substrate surface (hydrophilic or hydrophobic), nozzle viscosity (solution flow rate), working distance between the hopper and substrate, and spray-gun moving speed.

**Figure 12:**
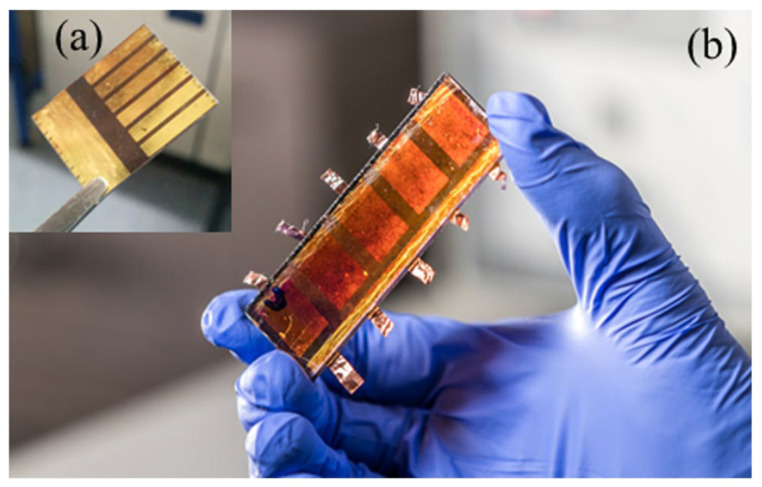
Comparison of (**a**) small- and (**b**) large-area PSC fabricated via spray coating. Reprinted from ref. [36].

Bishop et al. [36] reported the successful preparation of PSC devices on a 25 mm × 75 mm FTO substrate using spray coating. The active area of the PSCs was 1.5 cm^2^, as shown in Figure 12, and the efficiency reached a peak of 10.2%.

(e) Other Film-Coating Methods (Dip/Blade/Slot-die)

**Figure 13:**
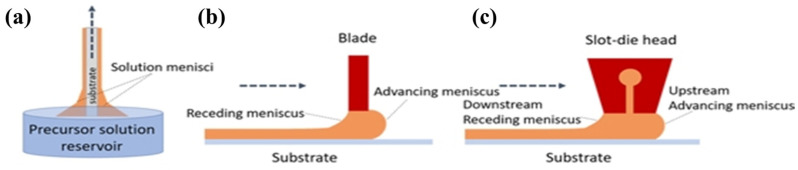
Schematic processes for film coating: (**a**) dip coating; (**b**) blade coating, and (**c**) slot-die coating. Reprinted from ref. [38].

**Figure 14:**
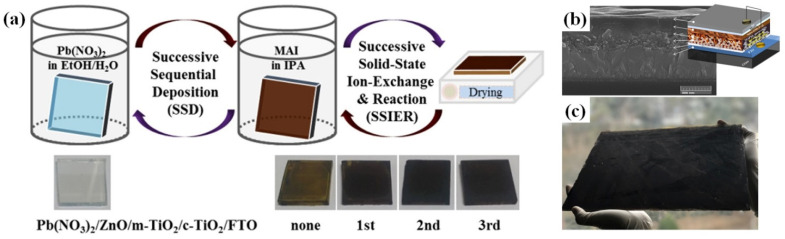
(**a**) Schematic diagram for the preparation of perovskite films using dipping of ZnO covered m-TiO_2_/c-TiO_2_/FTO substrates in aqueous Pb(NO_3_)_2_ and methylammonium iodide (CH_3_NH_3_I, MAI) solutions, (**b**) Cross-section SEM image of fabricated MAPbI_3_ perovskite solar cell device and (**c**) Photograph of the dip-coated perovskite solar cell via sequential successive sequential deposition (SSD) and successive solid-state ion-exchange and reaction (SSIER) deposition on large area glass substrate of 780 cm^2^ (30 cm  (Width) × 26 cm (Height)). Reprinted from ref. [37].

In addition, dip coating, blade coating, and slot-die coating have been used to fabricate solar cells (see Figure 13). Coating is a very simple, quick, and low-cost process for growing films in a solution. After the coating material was dissolved in the solvent, the substrate was placed inside the solution, where it remained for a specific time and then was removed. Once the solvent is dried by annealing, a film is formed on the substrate. Further, the film thickness can be controlled by changing the dipping speed, time, and temperature, as shown in Figure 14 [37].

Adan et al. [37] reported that all layers were deposited on FTO glass substrates using dip coating. After being coated for 600 s in the MAI solution, the MAPbI_3_ perovskite film was successfully formed on a large substrate (30 cm × 26 cm) with a PCE of 12.41% as shown in Figure 14. The blade (doctor blade or knife) coating is the most famous meniscus fabrication technique for large-area and low-cost film deposition [39]. Blade coatings have been used in organic material-based solar-cell devices such as PSCs. Blade coating was developed for screen printing and enables the designated patterns to be prepared while moving across the silkscreen on the substrate as shown in Figure 13b. Normal coating solutions can be used as the liquid type, which includes solvents or water. The substrate could be selected from a glass substrate to a conventional polymer, such as polyvinyl chloride (PVC), polyethylene terephthalate (PET), polytetrafluoroethylene, polyurethane, or other elastomers. For example, Grätzel et al. [40] reported that a modified TiO_2_ paste was prepared and used as an active layer in dye-synthesized solar cells (DSSCs) using blade coating. The blade has been used for compact TiO_2_ layers and mesoporous TiO_2_ in DSSCs. Once TiO_2_ powder is made into a fine powder with nanosized particles, the powder is mixed with a solvent, such as isopropyl alcohol, until it becomes thick. Then, a large amount of paste is obtained from the substrate and is pushed toward the designated area by pressing the blade with a small force, as shown in Figure 14b. After the film was uniformly spread on the substrate, the samples were dried until the solution was completely removed by heat annealing.

Slot-die coating is another film-coating technique for large-area deposition, wherein a coating solution is delivered onto the substrate by positioning a narrow gap between the slot and the surface of the substrate [38,39]. Compared to other traditional solution-coating methods, a major advantage is the uniform and very thin film across large areas of the substrate. As shown in Figure 13c, the slot-die coating system has a slot-die head, including a coating solution that is supplied from the solution container. The film thickness can be controlled by the flow rate of the solution and the slot-die head traveling time onto the substrate. If the slot-die head is not moving, then the film thickness can be controlled by the rotating speed of the coated substrate [39,41].

### 5.2. PV Module Manufacturing: From Cells to Modules

**Figure 15:**
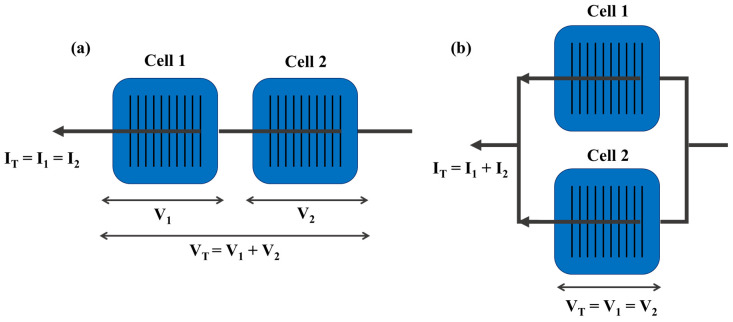
Schematics of (**a**) series- and (**b**) parallel-connected silicon solar cells. Reprinted with permission from ref. [44]. Copyright 2015 Elsevier.

From raw materials, the crystal-silicon wafer is fabricated by polishing and slicing Ingots grown using the Czochralski (CZ) method. Solar unit cells are fabricated on poly-/single-crystalline or mono silicon wafers [42]. Theoretically, a solar cell with silicon has at least 28% efficiency in terms of the unit cell. Commercial silicon-based PV devices have low voltage (0.6–0.7 V) and high current (~9 A). The total voltage increases as each cell is connected in series; for parallel combinations, the current increases without changing the voltage. As shown in Figure 15, PV modules consist of series- and parallel-connected solar cells (48–72 cells) [42]. After the modules are assembled with unit cells, the total efficiency decreases because of the problem of interconnecting to each module, including poor connection and failure. In addition, over 40% of solar modules failed to generate electricity because of interconnection breakage between individual solar cells [43].

Individual cell connection is performed after the discrete or monolithic processes are carried out. After the conversion materials are coated onto the conductive film, solar cells are divided into unit cells, and each cell is connected with a metal wire. In the monolithic process, a pathway is created by laser scribing and not cutting. In this process, all layers form on the same substrate [44,45].

The classic interconnections are monolithic, made by laser scribing for PV module arrays. Silicon-based solar cells are suitable for the design of monolithic modules. In particular, monolithically interconnected individual cells can be large-scale, light, and highly reliable without metal welding [46].

**Figure 16:**
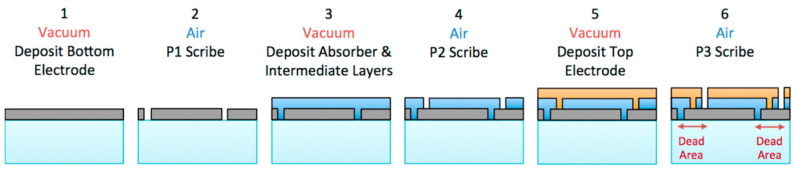
Fabrication for monolithic-cell integration using the conventional PV module, including an active area and a dead area consisting of the interconnections P1, P2, and P3. Reprinted with permission from ref. [47]. Copyright 2017 Elsevier.

PV modules with monolithic series connections are fabricated using three scribe steps, namely, P1, P2, and P3, via laser scribing, as shown in Figure 16. The laser scribing is sequentially lined next to each line.

The first scribe (P1) divides the conductive coating on the substrate into isolated stripes. After P1 scribing, all layers, including transparent conductive oxides (TCO), absorbance layer, and the back metal conductive layer, are independently formed on the substrate. The second scribe (P2) goes from the top to the metal conductive layer. After P2 scribing, the interconnecting path is provided, and each cell is connected to the bottom electrode (metal back contact). Finally, the third scribe (P3) cuts the top electrode (TCO) to isolate the cells and complete the fabrication into a series-interconnected structure. This three-scribe patterning process is repeated until the PV modules are completed (Figure 16). Further, unit cells have an active area and dead area in the PV module, as shown in the six steps in Figure 16. The area of P1–P3 is the non-generated section (or dead area), which often leads to energy loss in the modules. Photogeneration is performed only in the active area for power generation. The geometric fill factor (GFF) is defined as the ratio between the active area and the total area of the module and can be evaluated using the active area designated modules by means of the following equation [48]:

$$\mathrm{GFF}\;\left(\%\right)=\left(1-\;\frac{Dead\;Area}{Total\;Area}\right)\times 100$$

Organic-based cells such as the DSSC module can be designed as a monolithic type, and their active area ratio reaches over 90% [49]. However, because silicon is resistant to lasers, the GFF values should be close to almost 1.0.

## 6. Flexible Photovoltaics

### 6.1. Flexible Thin-Film c-Si Solar Cells

At the beginning of the PV era in the mid-1970s, crystalline silicon (c-Si) was used for solar cells, because it was an available abundant material that was relatively low cost, had a high carrier mobility, and could use well-established processing techniques [49]. However, in the early 1980s, crystalline silicon thin-film technology within the cell was fully recognized, which was the first time that high efficiency in thin films of silicon was achievable [50,51,52]. On a laboratory scale, Zhao fabricated stable silicon cells with 24.5% efficiency on magnetically confined CZ substrates under the standard global spectrum (100 mW/cm^2^) at 25 °C [53]. Whereas a 21.5% efficient thin-film solar cell has been achieved for a 47 μm-thick cell, the c-Si films become flexible at a thickness < 50 μm [54]. One of the most important advantages of increasing the c-Si thickness is the potential to reduce production costs and enable material reuse. PV devices based on silicon have dominated the market. In addition, the recombination in the bulk wafer has been limited to thin cells (~20 μm) with an *FF* approaching 0.9, and the open-circuit voltage *V_OC_* reaching 800 mV at room temperature [55]. To realize flexible thin-film c-Si solar cells that can be installed on intricate designs, uneven surfaces, and clothing, c-Si solar cells should be formed on suitable flexible substrates to replace the brittle and rigid substrates. Therefore, several methods have been investigated for the development of c-Si thin-film solar cells on flexible substrates.

(a) Transfer Technologies

**Figure 17:**
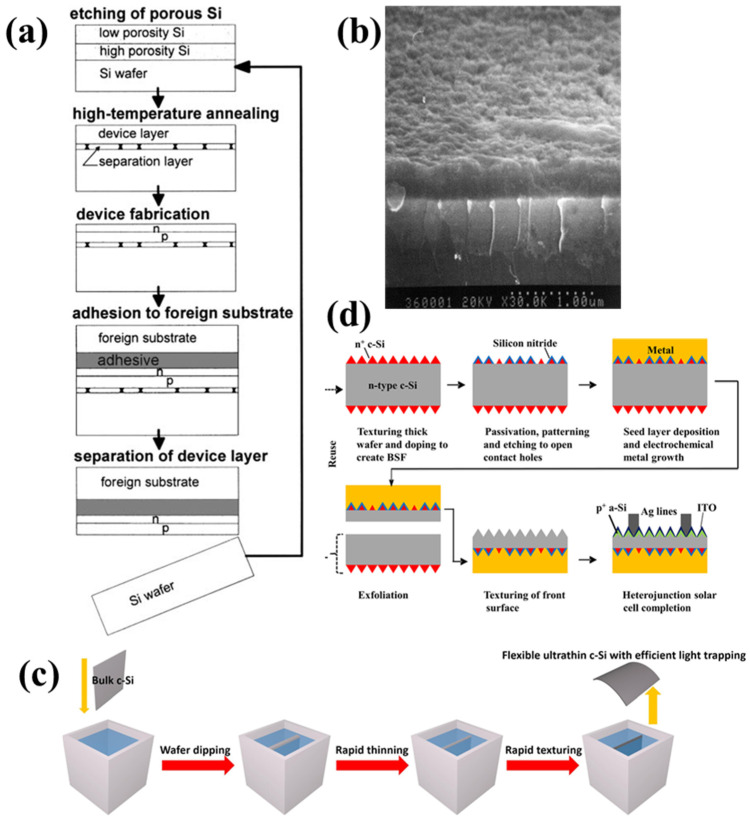
(**a**) Initial porous double-layer structure extending over a host wafer shown by a scanning electron micrograph. Reprinted with permission from ref. [57]. Copyright 1999 Springer Nature; (**b**) SEM image cross-section of the porous layer. Reprinted with permission from ref. [62]. Copyright 1997 Elsevier; (**c**) schematic for the ultrathin flexible c-Si fabrication using etching. Reprinted with permission from ref. [67]. Copyright 2018 Elsevier; (**d**) process flow for the solar cells on 25 μm-thick Si using exfoliation. Reprinted with permission from ref. [73]. Copyright 2013 AIP Publishing.

The transfer process is one of the most popular methods for fabricating c-Si solar cells. Single-crystalline films are produced by peeling off films from a single-crystalline Si wafer and transferring them to foreign substrates. This method was first reported by Werner, who transferred single-crystalline Si films onto a plastic foil and achieved efficiencies as high as 14% for cells with a thickness of 40 μm [56,57,58]. Figure 17a illustrates a host surface, where flexible c-Si solar cells start to form with the epitaxial growth of a monocrystalline Si thin film [56]. Several layers were first grown on the Si wafers. The Si thin-film cells were then detached from the silicon wafer and transferred onto a flexible polymer foil that served as a flexible foreign substrate. Thereafter, the Si wafer can be reprocessed for the next growth cycle; thus, waste of materials is limited. The restructuring process of the porous Si layer requires high-temperature treatment (>1050 °C) [59]. Higher deposition rates and high-quality films have been achieved by high-temperature deposition, leading to higher throughput [60]. The porous Si structure was reorganized because of the lower surface energy [61]. However, the annealing of the porous layer at high temperatures forms large voids. Hence, the porosity of the Si layer increases, as shown in Figure 17b [62]. The device is attached to a flexible foreign substrate from the host wafer by the direct application of a mechanical force. A 1 μm-thick layer of Al was evaporated onto the backside of the cell, and the back of the device served as the back contact layer [63]. A highly flexible 25 μm-thick c-Si film can be bent to a 2 mm curvature radius. In the bending experiments, an efficiency of 14.6% was independently confirmed after the film was transferred to a flexible plastic foil [64]. Solar cells prepared by transfer technology have achieved conversion efficiencies of up to 16% [63,65].

(b) Etching Method

Directly etching of the bulk wafer is the most common method among the various technologies developed for thinner Si wafers owing to a simple procedure that uses high-quality material [66]. Figure 17c shows a schematic of the chemical etching fabrication process for ultrathin flexible Si solar cells [67]. The wafer thickness was controlled by varying the etching time. To complete the fabrication, back-contact Ag material was deposited by sputtering to avoid strong parasitic absorption. Flexible PV modules of 15–20 μm thickness can achieve efficiencies in the range of 6–8% without back reflectors and retain their efficiency at a bending radius of 5 mm up to 200 cycles [68]. Cruz-Campa et al. fabricated flexible thin-film modules with an efficiency of 14.9% using an etching method that produced hexagonal Si segments [69]. Further, chemical etching of bulk c-Si employed a Cu-assisted method to obtain 45 μm-thick flexible solar cells that achieved a high efficiency of 17.3% [67].

(c) Exfoliation Method

Among the various ways to prepare thin-film c-Si solar cells, an exfoliation technique has been investigated recently to obtain ultrathin flexible monocrystalline Si substrates. The exfoliation technique is one of the promising techniques because of the simplicity of the process of cleaving. The process sequence begins with the deposition of Ni by sputtering as the stressor layer controlling the layer thickness [70]. In the exfoliation method, the thickness is controlled by varying the electroplated metal thickness and annealing process [71]. According to Niepelt, using Al-metallization layers as the stress-inducing element after the exfoliation process can provide a moderate cost-saving potential of 3–36% over that of other c-Si solar-cell production methods [72]. The flexible handle layer was attached to a Ni film covering without producing multiple cracks during the process. A 25 μm thin-film exfoliated monocrystalline Si solar cell was fabricated with 12.5% efficiency using an unoptimized single heterojunction cell process. In another study, a thick silicon foil formed by carefully controlling the conditions and texturing process achieved a high efficiency of up to 14.9% (Figure 17d) [73].

### 6.2. Flexible Thin-Film a-Si:H/μc-Si:H Solar Cells

Flexible hydrogenated amorphous (a-Si:H)/microcrystalline Si (μc-Si:H) thin-film solar cells have many advantages in terms of performance and large-scale production; these facilitate the scaled-up development of flexible solar cells other than flexible c-Si solar cells. They are either amorphous or microcrystalline and are deposited by plasma-enhanced chemical-vapor deposition (PECVD), including from 5 to 15% hydrogen atoms. Hydrogen atoms play an important role in the semiconductor films of flexible thin-film solar cells by passivating inherent defects [74]. Therefore, thin-film Si PV modules using a-Si:H/μc-Si:H absorbers prepared on flexible substrates, such as plastic, polymer, or plastic foil, are potential alternative candidates for flexible c-Si solar cells.

(a) Materials of flexible a-Si:H/μc-Si:H solar cells

**Figure 18:**
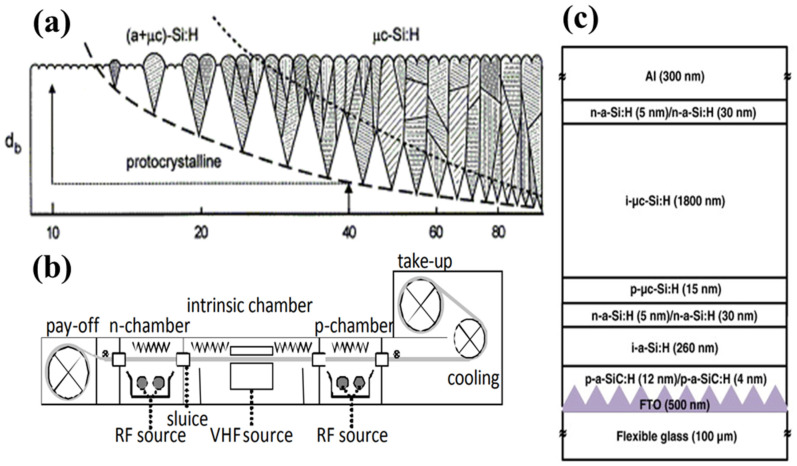
(**a**) Schematic of the structure of Si:H films prepared as a function of *R*. Reprinted with permission from ref. [81]. Copyright 2013 Elsevier; (**b**) cross section of the Flexicoat300 roll-to-roll PECVD system. Reprinted with permission from ref. [87]. Copyright 2010 EU PVSEC; (**c**) schematic cross-section of a PECVD-fabricated flexible a-Si:H/μc-Si:H double-junction solar cell with p-i-n configuration. Reprinted with permission from ref. [91]. Copyright 2014 Elsevier.

In a-Si:H/μc-Si:H single-junction solar cells, the active layer includes three Si layers, comprising n- and p-type doped layers, with an intrinsic (i) layer as the absorbed incident-light layer. The a-Si thin films were first prepared using the PECVD method created by Chitick [75]. The a-Si layer deposited from silane by PECVD was doped using either phosphine (PH_3_) or diborane (B_2_H_6_) as the n- and p-type dopants, respectively. Using this method for thin a-Si layers increased their conductivity by several orders of magnitude. Solar cells were first fabricated using a-Si:H with an efficiency of 2–5% in 1976 [76]. Moreover, high-quality solar cells using other methods have been investigated, such as low-pressure hot-wire-assisted chemical-vapor deposition [77,78,79] or very-high-frequency plasma deposition [80]. By changing the hydrogen dilution ratio (*R* = [H_2_]/[SiH_4_]) in the preparation process, different crystal phases can be obtained in the layers. For example, with low hydrogen dilution, amorphous layers are produced. As *R* increases, an amorphous phase and a low concentration of minuscule crystallites form; at a very high hydrogen dilution ratio, highly crystalline layers are formed. Figure 18a illustrates various film structures obtained using different PECVD parameters for films deposited on a glass substrate [81]. Single-chamber processes or multi-chamber deposition can be used for thin-film a-Si:H solar-cell production [82]. However, these processes have various drawbacks, such as cross-contamination of doping atoms [82,83,84,85,86]. The R2R production of thin-film Si solar cells introduces several advantages for the fabrication of lightweight, flexible, and low-cost products. Figure 18b shows the cross-section of an R2R PECVD system on flexible metal foils. Aken reported that a-Si solar cells fabricated by an R2R PECVD process achieved efficiencies of up to 6.6% [87]. The bandgap of the intrinsic μc-Si:H layer (1.1 eV) is lower than that of the intrinsic a-Si:H layers because of the crystalline phase of the materials [88]. Stable conversion efficiencies of 13.0% have been achieved using an a-Si-based material to absorb the green- and red-wavelength photons by controlling the ratio of silicon to germanium [89]. Thin-film silicon solar cells have extensively used tandem and multi-junction configurations, leading to the generation of various designs. First, in a simple a-Si:H/a-Si:H tandem configuration, the I layer of the subcells is thinner than that in single-junction cells with the same light absorption. Second, triple-junction cells based on a-Si–germanium alloys (a-Si:H/a-SiGe:H/a-SiGe:H) allow the bandgap of the individual cells to change. Third, the micromorph a-Si:H/μc-Si:H tandem cell uses a-Si on the top and μc-Si on the bottom [90].

(b) Structures of flexible a-Si:H/μc-Si:H solar cells

The first a-Si:H and μc-Si:H solar cells were fabricated by the p–i–n deposition sequence using a TCO-coated glass substrate. In this configuration, light enters the solar cell through the glass substrate, which functions as a contact layer. The p-layer is deposited on the top of the glass as the first semiconductor layer to allow the light to strike the thicker i-layer to generate charge carriers. Figure 18c illustrates the cross-sectional structure of flexible solar cells using the a-Si:H/μc-Si:H double junction, fabricated using the PECVD technique with a radio frequency of 13.56 MHz [92].

**Figure 19:**
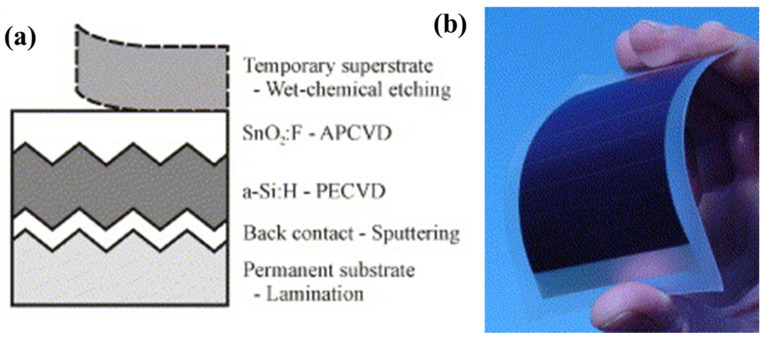
Temporary superstrate concept is shown (**a**) schematically and (**b**) in a photograph. Reprinted with permission from ref. [92]. Copyright 2007 Elsevier.

These flexible solar cells can achieve an efficiency of 8.0% and a stabilized efficiency of 7.1% without a back reflector layer. Donker first demonstrated flexible a-Si:H/μc-Si:H tandem modules in a superstrate configuration with efficiencies of up to 9.4% (Figure 19a,b) [92]. As another configuration, a substrate design using an n–i–p structure is generally suitable for flexible solar cells because of the advantages gained by using lightweight, strong substrates, such as stainless steel, PI, or PET. In this case, light enters the device through a semiconductor layer deposited as the final layer. Therefore, the fabricated device is different from superstrate-type devices, because of the modified deposition sequence [93]. By reducing the i-layer thickness from 400 to 250 nm, a 4.9% PCE was achieved using ZnO:Al/PET [94], and up to 5.9% efficiency was achieved by direct deposition of n–i–p-type a-Si cells fabricated at a low temperature (~100 °C) [95]. Thus, the mechanical strength and thermal stability of polymers are not as suitable as those of metal foils. In one study, a-Si:H/a-SiGe:H tandem solar cells were fabricated on plastic substrates to fabricate 40 × 80 cm cells with 9% efficiency [96]. Further, a flexible textured substrate was fabricated at 180 °C, demonstrating a high short-circuit current density (25.8 mA/cm^2^) with 8.1% efficiency [97].

Using this structure, Yan fabricated flexible a-Si:H-based triple-junction solar cells, achieving an efficiency of 15% [98]. Finding suitable polymers for solar-cell substrate materials is crucial, because they allow the fabrication of solar cells on plastics. A peel-and-stick method has been investigated on universal substrates without changing the conditions of material deposition or cell performance [99]. The solar cell was fabricated on a nickel-coated Si wafer under conventional conditions. The solar cell was attached to a thermal tape and soaked in water. Ultrathin flexible solar cells were obtained on the universal substrates after transfer.

### 6.3. Perovskite/c-Silicon Tandem Solar Cells

In addition to the advantages of PSCs in terms of efficiency, the limited absorption edge on the long wavelength (800 nm) restricts the development of efficiency beyond the Shockley–Queisser limit. Tandem PSCs are an innovative approach to ameliorate the disadvantages of perovskite absorbers. A perovskite tandem solar cell can be divided into 4-terminal (4T) and 2-terminal (2T) based on how the junctions are located between the top and bottom cells. In this paper, we focused on the evolution of monolithic perovskite/c-Si tandem solar cells designed in perovskite top cells and crystalline silicon (c-Si) bottom cells. Crystalline silicon (c-Si), which is classified into silicon homojunctions and silicon heterojunctions, is one of the most appropriate materials for high-efficiency PV devices because of their desirable intrinsic properties [100]. Therefore, monolithic perovskite/c-Si tandem cells can be divided into two different types: perovskite/homojunction and perovskite/heterojunction.

(a) Perovskite/Silicon Homojunction Solar Cells

**Figure 20:**
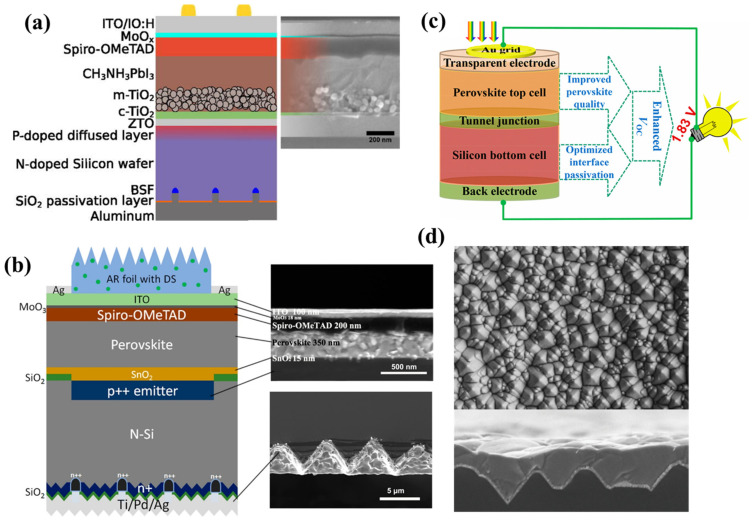
(**a**) Structure of the monolithic tandem cell with ZTO layer. Reprinted with permission from ref. [102]. Copyright 2016 AIP Publishing; (**b**) schematic of the perovskite/silicon solar cell and cross-sectional SEM image. Reprinted with permission from ref. [104]. Copyright 2019 American Chemical Society; (**c**) schematic of the device architecture of the perovskite/heterojunction solar cells. Reprinted with permission from ref. [105]. Copyright 2019 American Chemical Society; (**d**) top-view image of the textured c-Si surface and cross-section of the perovskite crystal layer. Reprinted with permission from ref. [109]. Copyright 2020 The American Association for the Advancement of Science.

A 2-terminal monolithic perovskite/silicon solar cell was first reported in 2015 and achieved a stable PCE of 13.7% with a *V_oc_* as high as 1.65 V [101]. However, this PCE value was still much lower than the record efficiency for PSCs, because of technological constraints under the perovskite-silicon tandem field. Further improvements can be achieved by using zinc tin oxide (ZTO) as a recombination layer to improve its electrical and optical stability at high temperatures. As shown in Figure 20a, a ZTO layer was directly sputtered onto the P-doped diffused layer, which increased the total transmittance at long wavelengths (800 nm) and the electron mobility from 21.8 to 35 cm^2^·V^−1^·s^−1^, which increased charge transportation with >16% efficiency [102]. This new homojunction c-Si cell architecture has been used with passivated and rear surfaces and rear-side texturing to process compact and mesoporous TiO_x_ at high temperatures. Consequently, these innovations boosted the efficiency of perovskite tandem solar cells up to 22.5% (steady state) with a *V_OC_* of 1.75 V on a 1 cm^2^ cell [103]. Recently, 23%-efficient monolithic perovskite/homojunction-silicon tandem solar cells have been reported on 4 cm^2^ cells by applying textured polydimethylsiloxane [104]. Figure 20b shows the schematic and cross-section of the perovskite top cell with the rear of the silicon bottom cells. The stability of perovskite tandem solar cells improved after 36 days of 8 h/day UV exposure, which is considered a significant improvement in the stability of PSCs.

(b) Perovskite/silicon heterojunction solar cells

Heterojunction solar cells (low-temperature tolerance < 250 °C) require a new approach to replace the high-temperature mesoporous or compact TiO_2_ layer. A 2-terminal heterojunction (SHJ) c-Si/perovskite tandem solar cell has been reported using a low-temperature-processed perovskite subcell that achieved an efficiency of 19.9% [105]. Figure 20c shows the structure of perovskite/silicon heterojunction solar cell, in which *V_OC_* for Cs-doped perovskite devices was due to the widened bandgap and reduced trap density. Using the same architecture of perovskite/heterojunction silicon tandem cells, Werner [106] investigated low-temperature NIR transparent perovskite cells with an efficiency of up to 25.2% with 0.25 cm^2^. Because of the developed rear side, QE in the near-infrared region could be improved, thus reducing the performance-limiting factor of perovskite/silicon tandem cells. The developed cesium formamidinium lead halide perovskite combined with an infrared-tuned silicon heterojunction bottom cell provides a stable and efficient perovskite tandem solar cell that has negligible parasitic absorption and good thermal stability when subjected to a 1000-hour test under harsh conditions [107]. Further improvements were achieved by investigating the influence of the current mismatch on the device performance [108]. The optimal perovskite thickness and top contact increased the *J_SC_* by approximately 19.5 mA cm^−2^, boosting the efficiency of tandem devices by up to 26%, with negligible hysteresis. Recently, an independently certified PCE of 25.7% for perovskite-silicon tandem solar cells was reported to have reduced the diffusion-length phase segregation [109]. The textured c-Si was fabricated by alkaline wet-chemical etching (Figure 20d), which changed the perovskite morphology and film thickness. The large grain sizes (up to 4 μm) of the perovskite layer covered the micrometer-sized pyramids c-silicon to achieve a uniform layer. Overall, the perovskite/homojunction tandem solar cells and perovskite/heterojunction solar cells are innovative approaches to deal with the instability and improve the efficiency of PSCs, which have been a great challenge for researchers to develop solar-energy systems.

## 7. Conclusions and Outlook

In this paper, we deal with energy-generation mechanisms and the developments and progress achieved in the areas of silicon-based flexible PV devices. Moreover, we introduced photovoltaic technologies for improving the efficiency of solar cells. To date, silicon-based solar cells have dominated the PV market, but they are no longer applicable for flexible PV applications, because they are heavy, brittle, and non-bendable. Despite all the challenges, harnessing new technologies for silicon-based flexible photovoltaic could provide an auspicious future such as pliable, low-temperature, and simple process of ultra-thin silicon wafers. For the device manufacturing methods, it is crucial to develop cost-effective methods to apply for large-scale modules such as roll-to-roll printing or spray coating method. For flexible photovoltaics, we reviewed flexible thin-film c-Si solar cells., flexible thin-film a-Si:H/μc-Si:H solar cells, and Perovskite/c-silicon tandem solar cells. Perovskite tandem solar cells are expected to dominate the market with high efficiency and long stability in the near future. In addition to establishing our own silicon technology, even though it has advantages in terms of large-scale modules, stability, and high efficiency, it extends the PV industry through integration with other technologies, such as organic solar cells. In addition, flexible silicon-based solar cells are low cost and have significant potential in applications such as roofs on houses, outdoor buildings, electronics, vehicles, and textiles.

## Data Availability

Not applicable.
